# Immune Responses of Asian Seabass *Lates calcarifer* to Dietary *Glycyrrhiza uralensis*

**DOI:** 10.3390/ani10091629

**Published:** 2020-09-11

**Authors:** Rui Yang, Mingyang Han, Zhengyi Fu, Yifu Wang, Wang Zhao, Gang Yu, Zhenhua Ma

**Affiliations:** 1 Tropical Aquaculture Research and Development Center, South China Sea Fisheries Research Institute, Chinese Academy of Fishery Sciences, Sanya 572018, China; janeyhn4321@yeah.net (R.Y.); myhan2019@163.com (M.H.); zhengyifu@163.com (Z.F.); 17854255640@163.com (Y.W.); zhaowang522@163.com (W.Z.); gyu0928@163.com (G.Y.); 2 Key Laboratory of South China Sea Fishery Resources Exploitation and Utilization, Ministry of Agriculture and Rural Affairs, Guangzhou 510300, China; 3 Sanya Tropical Fisheries Research Institute, Sanya 572018, China

**Keywords:** *Lates calcarifer*, *Glycyrrhiza uralensis*, immune-related genes, gene expression

## Abstract

**Simple Summary:**

Due to the fact of their low toxicity, small side effects, and little residue, increasing attention has been paid to herbs as environmentally friendly immunostimulants. Results from the present study indicate that adding *Glycyrrhiza uralensis* to the feed can improve the growth and survival of *Lates calcarifer* and significantly promote the expression of immune-related genes in the liver and head kidney of *Lates calcarifer*. The optimum inclusion level of *Glycyrrhiza uralensis* should be 1–3%.

**Abstract:**

To understand the impacts of dietary *Glycyrrhiza uralensis* on the immune responses of *Lates calcarifer*, the expression of immune-related genes including *crp*, *c-3*, *c-4*, *mtor*, *mlst-8*, *eif4e*, *hsp-70*, *hsp-90*, *il-8il-8*, *il-10*, *tgfβ1*, *tnf*, *ifn-γ1*, and *mxf* in *L. calcarifer* juveniles was evaluated in this study. Fish were fed experimental diets with *G. uralensis* levels of 0%, 1%, 3%, and 5% for 56 days. The results showed that dietary *G. uralensis* could improve the growth and survival of *L. calcarifer* and regulate the immune-related genes’ expression in *L. calcarifer*. Dietary *G. uralensis* significantly upregulated the expression level of *crp*, *mtor*, *hsp-90*, *c-3*, and *c-4* genes in the liver of *L. calcarifer*, while *hsp-70* gene expression was nearly downregulated. It did not upregulate the expression of *elf4e* and *mlst-8* in the 1% and 3% inclusion groups, but it was the exact opposite in the 5% inclusion group. *G. uralensis* significantly affected the expression of *il-8, il-10, tnf, ifn-γ1, mxf,* and *tgfβ1* in the head kidney of *L. calcarifer*. *G. uralensis* upregulated the expression of *tnf* and *tgfβ1* consistently, but *ifn-γ1* was at a low expression level. The expression of *il-8* and *il-10* was upregulated in the 1% group, while it was downregulated in the 5% group. The results from the present study indicate that dietary *G. uralensis* appeared to improve the immune function of *L. calcarifer*, and the optimum inclusion level should be between 1–3%.

## 1. Introduction

Asian seabass *Lates calcarifer* is widely distributed in the Indo–Pacific region. It is one of the most important marine aquaculture finfish in Australia and Asian countries, and the aquaculture of *L. calcarifer* increased to 76,842 tons in 2015 [1]. Because of its high nutritional value and rapid growth, *L. calcarifer* has become one of the main aquaculture species in southern China [2]. However, the outbreak of disease becomes a limiting factor in large-scale aquaculture of *L. calcarifer*. During aquaculture practices, high stocking density, grading, transporting, and environmental conditions may induce stress in fish, and this may cause disease outbreaks [3,4]. Diseases caused by bacteria, viruses or nutritional factors have been reported in farmed *L. calcarifer* such as scale drop syndrome (SDS) and megalocytiviral diseases [5,6]. Traditionally, antibiotics and chemotherapeutics administration are the major methods to treat the disease outbreaks, and these methods have a negative impact on the environment and fish. Recently, aquaculture specialists have made considerable progress in preventing and treating fish diseases through vaccines, probiotics, and immunostimulants [7,8,9].

Immunostimulants, which are feed additives derived from natural sources or synthetically, are capable of modulating the fish immune response and improving disease resistance. Therefore, people have begun to consider the immune stimulants used in the aquaculture industry [10,11]. Due to the low toxicity, small side effects, and little residue, increasing attention has been paid to herbs as environmentally friendly immunostimulants, and many studies have shown that herbs play a positive role in the prevention and treatment of fish diseases [11,12]. *Glycyrrhiza uralensis*, a leguminous flowering plant native to Asia, contains glycyrrhizic acid (GA) and other bioactive ingredients, and the extract of *G. uralensis* is widely used in China [13]. Evidence indicates that the extract of *G. uralensis* shows great potential as the immune stimulant in finfish aquaculture. Some reports have shown that feed supplemented with licorice extract or glycyrrhizic acid can improve the growth performance, immune response, stress resistance, and resistance to specified pathogens in fish species such as crucian (*Carassius auratus*), yellow croaker (*Larimichthys crocea*), and yellow catfish (*Pelteobagrus fulvidraco*) [14,15,16]. The addition of *G. uralensis* extract in the feed can promote the growth and lysozyme activity of yellow catfish and effectively reduce the mortality of fish infected with *Flavobacterium columnare*. Previous studies have explored the effects of plant or herbal, such as garlic, hirami lemon, and leaf meal, extracts as feed additives on the rearing performance of *L. calcarifer*, and positive effects in terms of growth and immunity have been observed (faster growth, higher disease resistance. and lower mortality rates) [4,9,10]. In this study, *G. uralensis* was used as the feed additive to test the immune responses of *L. calcarifer*. The expression of immune-related genes from fish liver and head kidney was evaluated, aiming to understand the immune responses of fish to immunostimulants and to provide more evidence for the availability of *G. uralensis* in fish feed.

## 2. Materials and Methods 

### 2.1. Experimental Diets

The feed formulas were designed with reference to the nutritional requirements [17] of *L. calcarifer* (Table 1). Four types of feed were designed with the content of *G. uralensis* as the gradient (0%, 1%, 3%, and 5%). The basal feed without *G. uralensis* (0%) was used as the control group. All raw materials were crushed and extruded into feed with a diameter of 2.0 mm. Afterward, all the experimental feeds were stored at −20 °C until further usage.

### 2.2. Animals

*L. calcarifer* juveniles were produced by Tropical Aquaculture Research and Development Center, South China Sea Fisheries Research Institute, Chinese Academy of Fishery Sciences. The average weight of experimental fish ranged 14.58 ± 2.15 g, and the average length was 9.53 ± 1.86 cm. 

This research was approved by the Animal Care and Use Committee of South China Sea Fisheries Research Institute, Chinese Academy of Fishery Sciences on 31 August 2018 (NO. 2018ZD01).

### 2.3. Experimental Design and Sampling

Before the experiment started, a total of 360 fish were randomly assigned to 12 tanks of 500 L (30 fish/tank) with a flow though system for a 14-day acclimation and fed with experimental diets. After acclimation, the feeding trail started and lasted 56 days. During the experimental period, fish feeding activity and death were observed and recorded, and the water quality was tested. The experiment was conducted in a circulation system and the water exchange rate was a 200% tank volume per day in each tank. The quality of water parameters was measured daily and maintained at a temperature of 27.5 ± 1.3 °C, a salinity of 32.5 ± 0.5‰, ammonia nitrogen < 0.01 mg/L, pH 7.5 ± 0.2, and nitrite nitrogen < 0.02 mg/L. The water quality parameters were maintained by adjusting the water exchange rate. Water was run through a mechanical filter and a biofilter and the water exchange occurred only when the water quality parameters exceeded the above ranges. The photoperiod was controlled at 14 h light and 10 h dark. Fish were fed ad libitum twice a day at 09:00 and 15:00. The feces and residual feeds were removed from the experimental tank daily by a siphon method. The residual feeds collected from each tank were dried and weighted.

At the end of the feeding trial, fish from each tank were counted for the final survival and were weighted to determine weight gain (WG). All the diets used in each tank were calculated to obtain feed intake (FI). Three fish from each tank were randomly collected after anaesthetizing with overdose of eugenol (7 mg/L eugenol, Shangchi Dental Material Co., Ltd., Changshu, China). Afterwards, fish were dissected on ice. Liver and kidney from each fish were stored in liquid nitrogen until further usage. Other fish growth performance indicators were calculated including specific growth rate (SGR), hepatosomatic index (HIS), and intraperitoneal fat ratio (IPF). The parameters were calculated as: WG = final body weight − initial body weight; FI = (feed consumed per tank/fish) / days, SGR = 100 × ((ln (final body weight) − ln (initial body weight)) /days; HIS = 100 × ((liver weight) / (whole-body weight)); IPF = 100 × ((intraperitoneal fat weight)/(whole-body weight)).

### 2.4. Gene Expression Analysis

The frozen tissue samples were homogenized in liquid nitrogen using a bioprep (Bioprep-24, Hangzhou Allsheng Instruments Co. Ltd., Hangzhou, China). The RNA extraction was performed according to the method described by Fu et al. [18]. The quantity of isolated RNA was determined by measuring their absorbance at 260 and 280 nm using an ND 5000 spectrophotometer (BioTeke Corporation, Beijing, China). Finally, the integrity of RNA was assessed using agarose gel (1%) electrophoresis. The cDNA was synthesized using the PrimeScript^®^ RT Master Mix (Perfect Real Time, Takara Biomedical Technology (Dalian) Co., Ltd., Dalian, China). The synthesized cDNA samples were stored at −20 °C until further use. The 10 μL of reaction: 2 μL 5 × PrimeScript^®^ RT Master Mix, RNA 500 ng, some Rnase Freed H_2_O (total volume was 10 μL). Reverse transcription reaction conditions: 37 °C, 30 min; 85 °C, 5 s.

The genes chosen for analysis by qPCR were selected from the *L. calcarifer* NCBI database (https://www.ncbi.nlm.nih.gov/). The Primer Premier 5 program (Premier Biosoft International, Palo Alto, CA, USA)was used for designing the primers of *crp, c-3, c-4, mtor, mlst-8, eif4e, hsp-70, hsp-90, il-8, il-10, tgfβ1, tnf, ifn-γ1, mxf,* and *β-actin* (Table 2). The direct and indirect effects of these gene transcription and translation products on fish immunity have been confirmed in numerous studies [19,20,21,22,23,24]. The qPCR was performed with the Real-Time qPCR analysis (Hangzhou Longgene Scientific Instrument Co., Ltd., Hangzhou, China) using SYBR Green (Tiangen Biotech Co., Ltd., Beijing, China) [18]. The 20 μL of reaction including 10 μL 2 × Real Universal PreMix, 0.6 μL 10 μM of each primer (10 μM), and 2 μL of diluted cDNA was initially denatured at 95 °C for 10 min and then amplified for 40 cycles (95 °C, 10 s, 58 °C, 20 s, and 72 °C 30 s). Each sample was subjected to qPCR for 3 times. At the end of each RT-qPCR cycle, the melting curve of the primer was analyzed to ensure that only specific products were obtained, and no primer dimer was formed. In addition, a negative control group without DNA a template was set to verify that the PCR process was not contaminated. The relative mRNA expression levels of the target genes were determined by the 2^−ΔΔ^Ct method and were normalized based on the level of housekeeping gene (*β-actin*). It has been verified that the reaction efficiency (E) was 90–105% and Pearson’s coefficients of determination (*R*^2^) > 0.98.

### 2.5. Statistical Analysis

The data were expressed as the mean ± standard deviation (SD). The software SPSS 19.0 (International Business Machines Corporation, Chicago, Illinois, USA) was used for statistical analysis, and one-way ANOVA and Least significant difference (LSD) test were used for inter-group comparison. The level of significance was set at *p* < 0.05. All data were tested for normality, homogeneity, and independence to satisfy the assumptions of ANOVA.

## 3. Results and Discussion

Compared with the control group, the weight gain rate and specific growth rate of each experimental group showed an upward trend, and both showed a significant increase at 5% of the experimental group (*p* < 0.05, Table 3). The survival rate increased with the increase of *G. uralensis* content. There was no significant difference in the feed intake and hepatosomatic index among all groups. Intraperitoneal fat ratio decreased with the increase of *G. uralensis* content, and there was a significant difference between the 5% group and other groups (*p* < 0.05).

**Figure 1:**
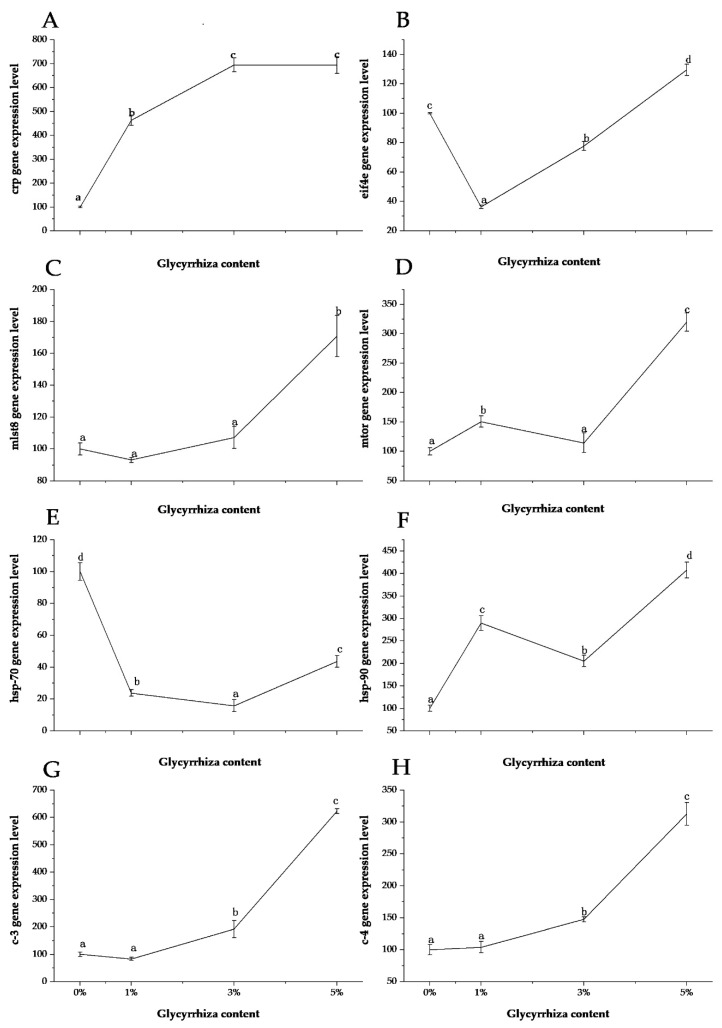
Relative expression of *Glycyrrhiza uralensis* on immune-related genes in the liver of *L. calcarifer*. (**A**) *crp*; (**B**) *eif4e*; (**C**) *mlst-8*; (**D**) *mtor*; (**E**) *hsp-70*; (**F**) *hsp-90*; (**G**) *c-3*; (**H**) *c-4*; Different superscript letters indicate significant differences among grades (*p <* 0.05) and the letters go from a to d, indicating that the level of expression increases. Error bars represent the standard error.

Dietary *G. uralensis* significantly affected the expression of immune-related genes, such as *crp eif4e mlst-8 mtor, hsp-70, hsp-90, c-3,* and *c-4*, in the liver of *L. calcarifer* (*p* < 0.05, Figure 1). Compared with the control group, the relative expression level of the *crp* gene in the experimental group was significantly upregulated (*p* < 0.05), and the expression levels in the 3% and 5% groups were significantly higher than in the 1% group (*p* < 0.05). The expression levels between the 3% and 5% groups were not significantly different (*p* > 0.05). The *eif4e* gene expression levels in the 1% and 3% grade were significantly downregulated (*p* < 0.05) and was significantly upregulated in the 5% group (*p* < 0.05). The expression levels of the *mlst-8* and *mtor* genes in the 1% and 3% groups were not significantly different from those in the control group (*p* > 0.05), but the relative expression levels in the 5% group were significantly upregulated (*p* < 0.05). *G. uralensis* had the opposite effect on genes *hsp-70* and *hsp-90*. It almost completely inhibited the expression of the *hsp-70* gene, but it had a significant upregulating effect on the *hsp-90* gene. *G. uralensis* had the same effect on genes *c-3* and *c-4*. The relative expression levels of *c-3* and *c-4* were not significantly different between the 1% group and the control group (*p* > 0.05) but were significantly upregulated in the other two groups (*p* < 0.05). Therefore, *G. uralensis* can promote the expression of most immune-related genes in the liver of *L. calcarifer*. Moreover, 5% *G. uralensis* can upregulate the expression of seven immune-related genes other than *hsp-70*.

**Figure 2:**
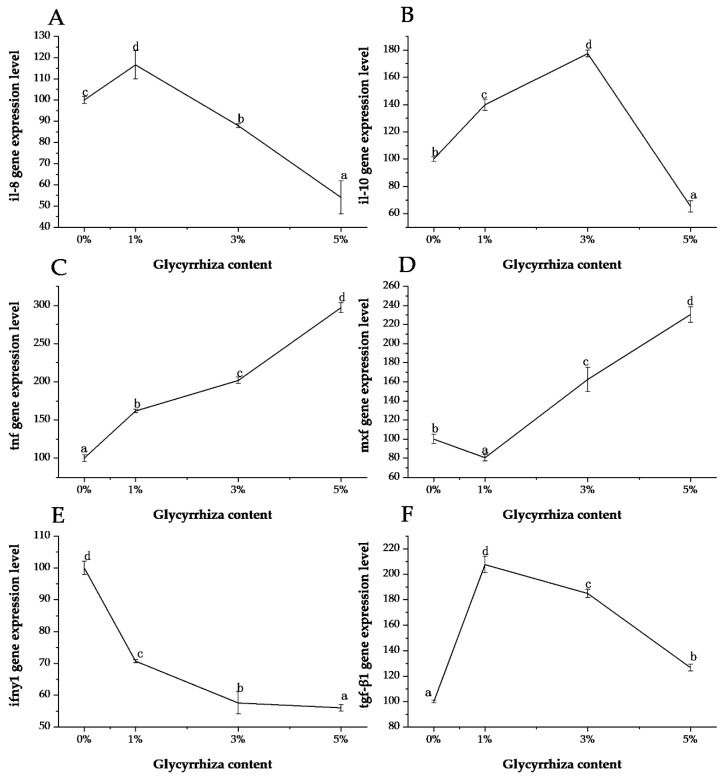
Relative expression of *Glycyrrhiza uralensis* on immune-related genes in the head kidney of *L. calcarifer.* (**A**) *il-8*; (**B**) *il-10*; (**C**) *tnf*; (**D**) *mxf*; (**E**) *ifn-γ1*; (**F**) *tgf-β1*; Different superscript letters indicate significant differences among grades (*p* < 0.05) and the letters go from a to d, indicating that the level of expression increases. Error bars represent theß standard error.

*G. uralensis* significantly affected the expression of kidney immune-related genes including *il-8, il-10, tnf, ifn-γ1, mxf*, and *tgfβ1* in *L. calcarifer* (*p* < 0.05, Figure 2). The content of 1% dietary *G. uralensis* could effectively upregulate the expression of *il-8* in the kidney of *L. calcarife*, but as the proportion of *G. uralensis* increased, the expression level of *il-8* was downregulated significantly (*p* < 0.05). The expression pattern of *il-10* was almost the same as that of *il-8*; 1–3% dietary *G. uralensis* could upregulate its expression but in excess, its relative expression was significantly downregulated (*p* < 0.05). For *tnf*, its relative expression was significantly upregulated with the increase of *G. uralensis* content (*p* < 0.05). The expression of *tnf* was the exact opposite to the expression of *ifn-γ1*; *G. uralensis* appears to have a strong inhibitory effect on *ifn-γ1*. A small amount of *G. uralensis* inhibited the expression of the *mxf* gene, but the expression was significantly upregulated when its content increased (*p* < 0.05). Dietary *G. uralensis* significantly upregulated the expression level of *tgfβ1* in fish (*p* < 0.05). The highest expression level of *tgfβ1* was observed in fish fed with 1% *G. uralensis* group (*p* < 0.05).

As a traditional Chinese herb, *G. uralensis* is mainly composed of triterpenoid saponins, flavonoids, coumarins, alkaloids, polysaccharides, and amino acids [25]. Its pharmacological effects mainly include antitumor [26], antiarrhythmic [27], antispasmolysis [28], antitussive [29], anti-inflammatory [30], antiviral [31], and immunoregulatory [32]. In aquaculture applications, *G. uralensis* plays a certain role in promoting the immunity and antioxidant capacity of aquatic animals. The addition of fermented licorice to *Epinephelus coioides* feed can reduce the damage to liver tissue and enhance antioxidant capacity thus improving the survival rate of fish under nitrite stress [33]. *G. uralensis* significantly improves the anti-stress ability of *Carassius auratus* [34]. It also increases the host’s resistance to *Aeromonas hydrophila* [34,35].

In terms of growth, the results of this study show that the addition of *G. uralensis* to feed has an obvious promoting effect on the growth of *L. calcarife* juvenile. Similarly, in the white shrimp *Litopenaeus vannamei*, the specific growth rate of the feed group with glycyrrhizin is significantly higher than that of the control group [36]. Feeding *G. uralensis* diets significantly increased (*p* < 0.05) growth performance and antioxidant and immune response in yellow perch *Perca flavescens* [37]. Glycyrrhetinic acid has been shown to increase the activity of fish digestive enzymes and to increase the expression of tumor necrosis factor (tnf-α) and lipoprotein lipase (lpl) to promote lipolysis for energy, thereby saving more protein for deposition for increased growth performance [15]. In this study, it was also found that the increase of *G. uralensis* content in the feed reduced the intraperitoneal fat ratio of *L. calcarife* juveniles. This suggests that dietary supplementation of licorice may enhance protein deposition in juveniles by promoting lipolysis, which might result in increased growth performance. Furthermore, in commercial fish, intraperitoneal fat is usually removed along with the viscera as an inedible portion, and it is generally undesirable, so feeding *G. uralensis* diets improves the product quality of *L. calcarife* to some extent [38]. In addition, the survival rate of the juveniles in the experimental group was significantly increased, which was a direct reflection of the effect of licorice on fish immunity. This result is similar to previous reports in yellow croaker [15] and yellow catfish [16]. After nitrite stress, the survival rate of *Epinephelus coioides* supplemented with fermented *G. uralensis* is also significantly improved [33]. Meanwhile, dietary *G. uralensis* supplementation did not decrease but slightly increased the intake of feed in the juveniles. This indicates that *G. uralensis* is a feasible feed additive.

Reactive protein (crp) is a phylogenetically highly conserved plasma protein, with homologs in vertebrates and many invertebrates, that participates in the systemic response to inflammation [21]. The crp is capable of specifically binding to and modulating the function of mononuclear phagocytes [39]. In this study, the expression of *crp* gene in the liver of *L. calcarifer* was upregulated after fish intake of *G. uralensis*. The relative expression levels of *crp* gene were highest in the 3% and 5% groups. Eukaryotic translation initiation factor 4E (eif4e) plays a central role in the recognition of the 7-methylguanosine-containing cap structure of mRNA and the formation of initiation complexes during protein synthesis. The gene *eif4e* exists in both phosphorylated and non-phosphorylated forms, and the primary site of phosphorylation has been identified. Previous studies have suggested that *eif4e* phosphorylation facilitates its participation in protein synthesis [40]. Our study showed that a small amount of *G. uralensis* had a certain inhibitory effect on *eif4e*, but a significant promoting effect was observed when the dietary *G. uralensis* inclusion level was over 5%. 

The mechanistic target of rapamycin (mtor) is the target of a molecule named rapamycin or sirolimus, which is a macrolide produced by *Streptomyces hygroscopicus* bacteria and that first gained attention because of its broad antiproliferative properties [41]. The mtor kinase nucleates two distinct protein complexes termed mtor-c1 and *mtor*-c2. The mtor-c1 responds to amino acids, stress, oxygen, energy, and growth factors and is acutely sensitive to rapamycin. The mtor-c2 responds to growth factors and regulates cell survival and metabolism as well as the cytoskeleton [42]. The mammalian lethal with SEC13 protein 8, is the binding protein of the target protein of rapamycin [43] and involved in both mtor-c1 and mtor-c2 [44]. In this study, the expression of *mtor* was upregulated in the 1% and 5% *G. uralensis* inclusion groups, and the highest expression level was observed in the 5% inclusion group. Similarly, the expression of *mlst-8* was upregulated significantly in the 5% group. It may suggest that 5% *G. uralensis* could promote the expression of *mtor* and its binding protein gene *mlst-8*.

Heat shock proteins (hsp) belong to the family of highly conserved cellular proteins present in all organisms that have been examined [45]. Hsp-70 and hsp-90 are the two main proteins in the heat shock protein family [23]. Hsp-70 is known to assist the folding of nascent polypeptide chains, act as a molecular chaperone, and mediate the repair and degradation of altered or denatured proteins [46]. Hsp-90 is active in supporting various components of the cytoskeleton and steroid hormone receptors [47]. Our results showed that *G. uralensis* had a consistent inhibitory effect on the expression of *hsp-70* gene in the liver of *L. calcarifer*, while it had the opposite effect on *hsp-90*, especially in the 5% inclusion group. Complement proteins c-3 and c-4 are also classified as acute phase reactants as their synthesis is upregulated during inflammation. In this study, low levels of *G. uralensis* did not affect the expression of *c-3* and *c-4* gene in the liver of *L. calcarifer*, while higher levels significantly upregulated their expression. This may suggest that higher dose *G. uralensis* can promote the expression of *c-3* and *c-4* effectively in *L. calcarifer*.

Interleukin-8 (il-8) is a chemokine that can activate neutrophils and has endogenous leukocyte chemokine and activation [48]. It is an important pleiotropic cytokine that mediates inflammatory responses and regulates the differentiation and proliferation of some immune cells. It mainly regulates the inflammatory response, which can not only inhibit mononuclear macrophages to release immune medium antigen presentation and cell phagocytosis [49]. In this study, the expression of *il-8* gene in the kidney of *L. calcarifer* in 1% groups was significantly higher than the other groups, and the expression level was downregulated as the content of *G. uralensis* increased. However, the highest level of *il-10* was found in the 3% group. Tumor necrosis factor (tnf), as a cytokine, it not only has cytotoxic effect on tumor cells, but also participates in a variety of pathophysiological processes such as antivirus, anti-infection, coagulation, fever and inflammation, shock, multi-organ failure and malignant fluid. Interferon (ifn) is a broad-spectrum antiviral glycoprotein secreted by recipient cells after viral infection of cells and the body or by nucleic acid bacterial endotoxin cytokinin. Ifn-γ1 is an important member of the ifn family, which also called Ⅱinterferon or immune interferon, mainly involved in inducing major histocompatibility antigen expression and immune regulation effect [50]. The results showed that the expression level of *tnf* was upregulated with the increase of content of *G. uralensis*, while the expression level of *ifn-γ1* was the opposite.

Transforming growth factor beta (tgfβ) family is a kind of superfamily polypeptide which has the function of regulating cell growth and differentiation, and tgf-β1 is a member of this family [51]. In this study, we could clearly see that the relative expression level of the *tgfβ1* gene in the kidney of *L. calcarifer* with *G. uralensis* was significantly higher than that of the control group. Myxovirus resistance (mx) is an antiviral protein that can be activated by ifn-I. Mx proteins belong to the dynamin superfamily and contain a tripartite guanosine triphosphate (GTP) binding domain which is essential for the antiviral activity [52,53]. In our study, adding 3–5% *G. uralensis* could significantly upregulate the expression of *mxf* gene.

## 4. Conclusions

In summary, dietary *G. uralensis* significantly improved growth performance and promoted the expression of immune-related genes in the liver and the kidney of *L. calcarifer*. Dietary *G. uralensis* can significantly upregulate the expression level of *crp, mtor, hsp-90, c-3*, and *c-4* genes in fish liver, and significantly affected the expression of *il-8, il-10, tnf, ifn-γ1, mxf*, and *tgfβ1* in fish kidney. Results from the present study indicated that dietary *G. uralensis* may improve the immune function of *L. calcarifer*, and the optimum inclusion level should be 1–3%. Adding *G. uralensis* to the feed will help to improve the growth, survival, and immunity of *L. calcarife*.

## Figures and Tables

**Table 1:** Feed formula and ingredient list.

Ingredients	0% Control Group	1% Test Group	3% Test Group	5% Test Group
Fish Meal	50	50	50	50
Flour	23	22	20	18
Soybean Meal	12.9	12.9	12.9	12.9
Vitamin Premix	0.5	0.5	0.5	0.5
Mineral Premix	0.5	0.5	0.5	0.5
Fish Oil	13	13	13	13
Glycyrrhiza Meal	0	1	3	5
Choline Chloride	0.1	0.1	0.1	0.1
***Dry Ingredients***				
Crude Protein	41.44	41.31	41.06	40.81
Crude Lipid	17.53	17.51	17.46	17.41
Crude Ash	9.26	9.22	9.13	9.05
Total Energy	20.28	20.12	19.79	19.46

Notes: (1) vitamin premix (mg or IU·kg^−1^): vitamin A 900,000 IU, vitamin D 250,000 IU, vitamin K_3_ 60 IU, vitamin E 50 IU, vitamin B_1_ 320 mg, vitamin B_2_ 1090 mg, vitamin B_5_ 2000 mg, vitamin B_6_ 500 mg, vitamin B_12_ 116 mg, vitamin C 5000 mg, niacin 40 mg, folic acid 5 mg, calcium pantothenate 20 mg, phaseomannite 150 mg, biotin 0.2 mg; (2) mineral premix (g·100 g^−1^): MgSO_4_·7H_2_O 3.0, KCl 0.7, KI 0.015, ZnSO_4_·7H_2_O 0.14, MnSO_4_·4H_2_O 0.03, CuCl_2_ 0.05, CoCl·6H_2_O 0.005, FeSO_4_·7H_2_O 0.15, KH_2_PO_4_·H_2_O 45.0, CaCl_2_ 28.0. The dietary energy was calculated as protein: 23.64 MJ·kg^−1^, lipid: 39.54 MJ·kg^−1^, carbohydrate: 17.15 MJ·kg^−1^.

**Table 2:** Primer of the immune-related genes in *L. calcarifer* used in qPCR.

Sample	Gene Abbreviation	Primer Sequence (5′–3′)	Amplicon Size (bp)	Accession No.
Head Kidney	*il-8*	F: TCTGACTGTTCCTGAGGCTATC	92	XM_018695863
		R: GACGTCCAATGGGCTTTCT		
	*il-10*	F: TGCTGCCGTTTTGTGGAG	194	XM_018686737
		R: ACCGTGCTCAGGTAAAAGTCC		
	*tgfβ1*	F: TACCTCGCTTCCCGTTTC	105	XM_018665504
		R: CTGCTCATCCTCAGTCCCTC		
	*tnf*	F: AAGGACTCCGCTGAGAAAAC	241	XM_018699809
		R: TGAACGATGCCTGGCTGTA		
	*ifn-γ1*	F: TACCAGGAGCAGGACAAGC	134	NM_001360734
		R: TCGTCAGGCAGCGAACTT		
	*mxf*	F: GGTGGACAAAGGCACAGAA	215	AY821518
		R: GTTTAGGAACGGTGGCATG		
Liver	*crp*	F: ACCGAACTGAAGACCACGAT	106	HQ652974
		R: TGGGGCACCTCAAACAAA		
	*c-3*	F: AAATGCTGCCATCGTTCC	175	XM_018679796
		R: CCAGTGACCTTCAGACCAAA		
	*c-4*	F: CGAGGTTGAACGAAAAGGAC	97	XM_018688206
		R: CACAGCAAGCAAAGCCACT		
	*mtor*	F: GTTTCTTCCGCTCCATTTC	110	XM_018675222
		R: CAGGGCTTCATTCACTTCA		
	*mlst-8*	F: TGATTCAACACTATTAGCCACA	212	XM_018687802
		R: TTTCCACGCACCACAGG		
	*eif4e*	F: TGACGACTACAGCGATGAT	183	XM_018697729
		R: GTGTCTGCGTGGGATTG		
	*hsp-70*	F: CTGGAGTCCTACGCTTTCAA	204	HQ646109
		R: CTTGCTGATGATGGGGTTAC		
	*hsp-90*	F: ACGATGATGAGCAGTATGCC	201	XM018661637
		R: CAAACAGGGTGATGGGGTA		
Head Kidney and Liver	*β-actin*	F: AACCAAACGCCCAACAACT	112	XM_018667666
		R: ATAACTGAAGCCATGCCAATG		

Note: C-reactive protein (*crp*), complement *c-3* (*c-3*), complement *c-4* (*c-4*), mechanistic target of rapamycin (*mtor*), mammalian lethal with SEC13 protein 8 (*mlst-8*), eukaryotic translation initiation factor 4E (*eif4e*), heat shock cognate 70 kDa protein (*hsp-70*), heat shock cognate 90 kDa protein (*hsp-90*), interleukin-8 (*il-8*), interleukin-10 (*il-10*), transforming growth factor beta-1 (*tgfβ1*), tumor necrosis factor (*tnf*), interferon gamma 1 (*ifn-γ1*), and myxovirus resistance factor (*mxf*) genes.

**Table 3:** Effects of different levels of *Glycyrrhiza uralensis* in feed on the growth performance of *L. calcarifer*.

Productivity Index	Experimental Diets			
	0%	1%	3%	5%
WG (g fish^−1^)	10.74 ± 0.35 ^a^	14.91 ± 0.06 ^b^	13.91 ± 3.34 ^b^	16.33 ± 2.47 ^b^
SGR (% d^−1^)	1.04 ± 0.05 ^a^	1.31 ± 0.08 ^ab^	1.23 ± 0.21 ^ab^	1.37 ± 0.21 ^b^
Survival (%)	73.33 ± 16.67 ^a^	79.25 ± 11.15 ^ab^	98.89 ± 1.92 ^b^	95.56 ± 5.09 ^b^
FI (g fish^−1^·d^−1^)	0.76 ± 0.06	0.86 ± 0.06	0.84 ± 0.08	0.90 ± 0.09
HIS (%)	2.32 ± 0.28	2.12 ± 0.31	2.49 ± 0.43	1.86 ± 0.32
IPF (%)	5.76 ± 1.42 ^b^	5.51 ± 0.50 ^b^	4.09 ± 1.35 ^b^	2.90 ± 1.01 ^a^

Note: WG, weight gain; SGR, specific growth rate; FI, feed intake; HIS, hepatosomatic index; IPF, intraperitoneal fat ratio. Different superscript letters indicate significant differences among grades (*p* < 0.05) and the letters go from a to b, indicating that the value increases.
